# Activation of the Nrf2 response by intrinsic hepatotoxic drugs correlates with suppression of NF-κB activation and sensitizes toward TNFα-induced cytotoxicity

**DOI:** 10.1007/s00204-015-1536-3

**Published:** 2015-05-31

**Authors:** Bram Herpers, Steven Wink, Lisa Fredriksson, Zi Di, Giel Hendriks, Harry Vrieling, Hans de Bont, Bob van de Water

**Affiliations:** Division of Toxicology, Leiden Academic Center for Drug Research, Leiden University, Einsteinweg 55, 2333 CC Leiden, The Netherlands; Department of Human Genetics, Leiden University Medical Center, Leiden, The Netherlands

**Keywords:** Drug-induced liver injury, Live-cell imaging, Nrf2 activation, Oxidative stress, NF-κB signaling

## Abstract

Drug-induced liver injury (DILI) is an important problem both in the clinic and in the development of new safer medicines. Two pivotal adaptation and survival responses to adverse drug reactions are oxidative stress and cytokine signaling based on the activation of the transcription factors Nrf2 and NF-κB, respectively. Here, we systematically investigated Nrf2 and NF-κB signaling upon DILI-related drug exposure. Transcriptomics analyses of 90 DILI compounds in primary human hepatocytes revealed that a strong Nrf2 activation is associated with a suppression of endogenous NF-κB activity. These responses were translated into quantitative high-content live-cell imaging of induction of a selective Nrf2 target, GFP-tagged Srxn1, and the altered nuclear translocation dynamics of a subunit of NF-κB, GFP-tagged p65, upon TNFR signaling induced by TNFα using HepG2 cells. Strong activation of GFP-Srxn1 expression by DILI compounds typically correlated with suppression of NF-κB nuclear translocation, yet reversely, activation of NF-κB by TNFα did not affect the Nrf2 response. DILI compounds that provided strong Nrf2 activation, including diclofenac, carbamazepine and ketoconazole, sensitized toward TNFα-mediated cytotoxicity. This was related to an adaptive primary protective response of Nrf2, since loss of Nrf2 enhanced this cytotoxic synergy with TNFα, while KEAP1 downregulation was cytoprotective. These data indicate that both Nrf2 and NF-κB signaling may be pivotal in the regulation of DILI. We propose that the NF-κB-inhibiting effects that coincide with a strong Nrf2 stress response likely sensitize liver cells to pro-apoptotic signaling cascades induced by intrinsic cytotoxic pro-inflammatory cytokines.

**Electronic supplementary material:**

The online version of this article (doi:10.1007/s00204-015-1536-3) contains supplementary material, which is available to authorized users.

## Introduction

Drug safety issues that lead to drug-induced liver injury (DILI) are the major reason for drug-related hospitalizations and drug withdrawals. Often with no overt changes in hepatocellular toxicity parameters (e.g., rise in alanine or aspartate aminotransferase (ALT/AST) levels or increased total bilirubin) found in preclinical settings, drugs are (unknowingly) safely marketed until more than 1 in 10,000 drug users demonstrate signs of liver failure (Kaplowitz 2005). Novel, predictive systems for DILI based on mechanistic understanding will be essential to pave the way forward for improved drug safety assessment.

The common notion around DILI is that drugs affect the intracellular biochemistry of liver cells, elicited by either the parent drug, its metabolites, or the metabolic shift the drug conveys upon uptake (Han et al. 2013; Kaplowitz 2005). Although often idiosyncratic, there is a need to understand the key events that are critical mechanistic determinants of human DILI. Perturbations of immune-mediated signaling seem an important event in DILI (Steuerwald et al. 2013). In particular, TNFα-mediated signaling seems an important contributor to sensitize liver cells to drug-induced hepatocyte toxicity both in vitro (Cosgrove et al. 2009) and in vivo (Shaw et al. 2007). TNFα mediates intracellular signaling through activation of NF-κB transcription factor (Mercurio et al. 1997). NF-κB transiently translocates to the nucleus to activate downstream (cytoprotective) target genes including chemokines, inhibitor of apoptosis protein family members (IAPs) and anti-apoptotic Bcl2 family members (Liu et al. 1996). We demonstrated that for diclofenac (DCF), the synergy with TNFα to kill hepatocytes is directly related to inhibition of NF-κB nuclear translocation and activation and that inhibition of NF-κB signaling sensitizes toward cytotoxicity caused by DCF (Fredriksson et al. 2011).

Bioactivation of drugs contributes to the formation of reactive metabolites which is shown to be a risk factor in DILI (Leung et al. 2012). These reactive metabolites typically provoke a cellular oxidative stress environment, thereby initiating the stabilization and activation of the transcription factor Nrf2 (Li et al. 2005). Subsequent downstream target gene activation by Nrf2 contributes to adaptation and protection of cells against oxidative stress. Likewise, Nrf2 deletion in the liver severely increases the sensitivity toward drug-induced liver failure (Liu et al. 2010, 2013). In some studies, it has been shown that Nrf2 activation can act to suppress NF-κB-based immune signaling responses (Chen et al. 2006), which is interesting as this would suggest that Nrf2 could be involved in NF-κB suppression in certain situations including DILI. So far, there is no systematic evaluation on the relationship between Nrf2 and NF-κB activation in DILI.

Here, we investigated whether drugs with known risk of DILI invoke specific cellular stress and defense pathways (NF-κB and Nrf2) and if these can aid in predicting the degree of drug toxicity and whether associations between these pathways exist. We investigated the transcriptional response to 90 DILI-associated drugs as well as several cytokines/growth factors in primary human hepatocytes (PHH) at multiple concentrations and time points, based on publicly available data (Uehara et al. 2010). To translate these findings to high-throughput approaches, we established novel GFP-based reporter cell lines amenable for high-content high-throughput live-cell imaging to quantitatively assess Nrf2 and NF-κB activation on a cell-to-cell basis. Our combined data indicate that the degree of oxidative stress in liver cells negatively correlates with NF-κB activity and that the inability to adequately respond to inflammatory responses upon drug exposure predisposes liver cells toward cell death. We propose that our integration of live-cell high-content imaging models to determine Nrf2 and NF-κB activation as well as cytotoxicity is likely to contribute to improving the discrimination of novel drug entities that are intrinsically at risk of DILI.

## Materials and methods

### Reagents

All drugs were acquired from Sigma-Aldrich and freshly dissolved in DMSO, except for menadione (MEN) and naproxen (NPX) (in PBS). Human TNFα was purchased from R&D systems and stored as 10 μg/mL in 0.1 % BSA in PBS aliquots.

### Cell culture

Human hepatoma HepG2 cells were acquired from ATCC (clone HB8065) and maintained and exposed to drugs in DMEM high glucose supplemented with 10 % (v/v) FBS, 25 U/mL penicillin and 25 μg/mL streptomycin. The cells were used between passage 5 and 20. For live-cell imaging, the cells were seeded in Greiner black μ-clear 96-well plates, at 20,000 cells per well.

### Gene expression analysis

CEL files were downloaded from the Open TG-GATEs database for all DILI-related compounds (see Supplementary Table 1): “Toxicogenomics Project and Toxicogenomics Informatics Project under CC Attribution-Share Alike 2.1 Japan” http://dbarchive.biosciencedbc.jp/en/open-tggates/desc.html. Probe annotation was performed using the hthgu133pluspmhsentrezg.db package version 17.1.0, and probe mapping was performed with hthgu133pluspmhsentrezgcdf downloaded from NuGO (http://nmg-r.bioinformatics.nl/NuGO_R.html). Probe-wise background correction (robust multi-array average expression measure), between-array normalization within each treatment group (quantile normalization) and probe set summaries (median polish algorithm) were calculated with the rma function of the Affy package (Affy package, version 1.38.1) (Irizarry et al. 2003). The normalized data were statistically analyzed for differential gene expression using a linear model with coefficients for each experimental group within a treatment group (Wolfinger et al. 2001).

**Table 1:** Summary of DILI compound modulation of Nrf2 and NF-κB signaling and onset of DILI compound/TNFα cytotoxic synergy

Drug name	Abbreviation	Function	DILI label/score	DILI classification	DILI type	DILI concern	Nrf2 response assay (fold induction)	NF-κB oscillation delay upon TNFα	Apoptosis assay (+last 16 h TNFα)	Apoptosis effect of TNFα (% increase)	Nrf2 transcripts up and NF-κB transcripts down
Troglitazone	TGZ	Antidiabetic	N.A.	N.A.	Acute—cholestatic injury	Most	1.3×	2 min	1.1 % (2.6 %)	1.5	N.A
Isoniazid	INH	Antimycobacterial drug	B.W. 8	Fatal hepatotoxicity	Acute—hepatocellular injury	Most	1.0×	2 min	2.8 % (3.3 %)	0.5	2–9
Ofloxacin	OFX	Antibiotic	N.A.	N.A.	Acute—hepatocellular injury	Less	1.1×	8 min	2.3 % (3.2 %)	0.9	N.A
Simvastatin	SN	Antihyperlipidemic	W/P 3	Liver aminotransferases increase	Acute—hepatocellular injury	Less	1.1×	2 min	2.5 % (3.6 %)	1.1	2–19
Naproxen	NPX	NSAID	W/P 3	Liver aminotransferases increase	Acute—cholestatic injury	Less	1.8×	4 min	2.1 % (2.3 %)	0.2	2–4
Methotrexate	MTX	Antineoplastic agent	B.W. 3	Liver aminotransferases increase	Chronic—microvesicular steatosis	Less	3.3×	9 min	1.9 % (1.9 %)	0	N.A
Amiodarone	AMI	Antiarrhythmic agent	B.W. 8	Fatal hepatotoxicity	Chronic—steatohepatitis	Most	1.9×	22 min	5.8 % (9.0 %)	3.2	0–0
Acetaminophen	APAP	Analgesic and antipyretic	W/P 5	Jaundice	Acute—hepatocellular injury	Most	4.0×	4 min	2.5 % (2.5 %)	0	12–29
3′-Hydroxyacetanilide	AMAP	Regioisomer of paracetamol	N.A.	N.A.	N.A.	Less	4.0×	4 min	3.1 % (3.4 %)	0.3	N.A
Nitrofurantoin	NTF	Antibacterial	W/P 8	Fatal hepatotoxicity	Chronic—autoimmune hepatitis	Most	4.6×	29 min	2.9 % (3.6 %)	0.7	15–23
Nefazodone	NFZ	Antidepressant	B.W. 8	Fatal hepatotoxicity	Acute—hepatocellular injury	Most	4.8×	22 min	3.5 % (6.8 %)	3.3	13–20
Clozapine	CLZ	Antipsychotic drug	W/P 25	Cholestasis; steatohepatitis	Acute—cholestatic injury	Most	4.6×	12 min	4.0 % (7.7 %)	3.7	1–12
Carbamazepine	CBZ	Antiepileptic drug	W/P 7	Acute Liver Failure	Acute—cholestatic injury	Most	4.1×	20 min	3.9 % (22.5 %)	18.6	4–9
Diclofenac	DCF	NSAID	W/P 8	Fatal hepatotoxicity	Acute—hepatocellular injury	Most	6.7×	26 min	4.5 % (14.2 %)	9.7	12–23
Ketoconazole	KTZ	Antifungal antibiotic	B.W. 8	Fatal hepatotoxicity	Acute—hepatocellular injury	Most	8.3×	26 min	5.0 % (8.1 %)	3.1	9–17

Full names, abbreviations and function of the drugs chosen for this study. The DILI classification was derived from Chen et al. (2011). The overall results from the current study are summarized as fold induction of the Srxn1-GFP intensity compared to control (Nrf2 response), timing of the GFP-p65 assay, focusing on the delay in the second nuclear translocation event upon TNFα exposure (NF-κB response) and percentage of dead cells as observed by the Annexin-V live assay (including TNFα-enhanced cell death)

A contrast analysis was applied to compare each exposure with the corresponding vehicle control. For hypothesis testing, the empirical Bayes statistics for differential expression was used followed by an implementation of the multiple testing correction of Benjamini and Hochberg (1990) using the LIMMA package (Smyth et al. 2005).

### Cluster analysis of oxidative stress and inflammation-regulated gene sets

A gene set for oxidative stress and a gene set for inflammatory signaling were generated using several databases (see Supplementary Fig 1). From Ingenuity Pathway Analysis (version 18841524), the genes present in the following pathways were extracted: NRF2-mediated oxidative stress response, death receptor signaling, NF-κB signaling, TNFR1 signaling, TNFR2 signaling and Toll-like receptor signaling. From the Gene Ontology Project (Ashburner et al. 2000), genes associated with the following terms were obtained using AmiGO 2 version 2.2.0 (Carbon et al. 2009): response to oxidative stress (GO:0006979) for oxidative stress and regulation of inflammatory response (GO:0050727) for inflammatory signaling. Both queries were performed with filters evidence-type closure set to “experimental evidence” and taxon closure label set to “Homo sapiens.”

From the Molecular Signatures Database (MSigDB) (Liberzon et al. 2011), for oxidative stress the following gene sets from BioCarta were used: BIOCARTA NRF2 PATHWAY and for inflammatory signaling BIOCARTA NFKB PATHWAY, BIOCARTA DEATH PATHWAY, BIOCARTA TNFR1 PATHWAY, BIOCARTA TNFR2 PATHWAY and BIOCARTA TOLL PATHWAY.

From Kyoto Encyclopedia of Genes and Genomes (KEGG Release 71.0, July 1, 2014): (Kanehisa et al. 2014) the pathways NF-κ B signaling pathway, TNF signaling pathway and Toll-like receptor signaling pathway were used for inflammatory signaling. No entry for Nrf2 or oxidative stress was found. From Reactome (version 48) (Croft et al. 2014) the pathways innate immune system and detoxification of reactive oxygen species (ROS) were used for inflammatory signaling and oxidative stress signaling, respectively. From “TRANSFAC^®^ (www.biobase-international.com/transcription-factor-binding-sites) from BIOBASE Corporation” (Qian et al. 2006), the genes bound by factor NFE2L2 and RELA were used for oxidative stress and inflammatory signaling, respectively.

From all databases, a total of 490 and 175 unique genes were obtained for inflammatory and oxidative stress signaling, respectively. As a next step to determine whether the selected genes are actively transcribed in PHH of the TG-GATEs dataset, another selection step was performed using the oxidative stress model compounds: di-ethyl maleate (DEM) and butylated hydroxyanisole (BHA), and inflammatory model treatments: TNFα, LPS and interleukin-1β; both for the high-dose 8- and 24-h data. The oxidative stress gene set was filtered based on a multiple-testing-corrected *p* value of 0.05, minimum average expression of 5 (log2) and a minimum absolute log2-fold change of 1.5 within the oxidative stress model compound subset resulting in 55 genes. The inflammatory signaling gene set was filtered based on a multiple-testing-corrected *p* value of 0.05, minimum average expression of 5 (log2) and a minimum absolute log2-fold change of 2 within the inflammatory signaling model treatment subset resulting in 82 genes. The log2-fold change values for all DILI treatments and controls were gathered followed by Manhattan distance measure and ward clustering using the NMF package (version 0.20.5) (Gaujoux and Seoighe 2010). Different log2-fold change threshold values were used to obtain more similar gene set sizes.

The DILI score annotation was adapted from the manual literature survey performed by Astrazeneca (Garside et al. 2014). The DILI concern and SeverityScore were largely based on a text mining study of FDA labels (Chen et al. 2011).

### Ingenuity Pathway Analysis

Differentially expressed genes for all DILI compounds in the TG-GATEs dataset were selected based on a minimal log2-fold change of 1.3 (fold change of 2.5 × with respect to matched control), a maximum multiple-testing-corrected *p* value of 0.05 and a minimum average log2 expression of 7 within the treatment groups (Supplementary Fig 1). Classification of the selected genes according to their biological and toxicological functions was generated through the use of QIAGEN’s Ingenuity Pathway Analysis (IPA^®^, QIAGEN Redwood City, www.qiagen.com/ingenuity), which finds associated canonical pathways based on the selected gene sets. *p* values are calculated using right-tailed Fisher exact test and represented as −log10 (*p* values). The *p* values were extracted for the “Nrf2-mediated oxidative stress response” pathway representing oxidative stress, and as representation for “inflammatory signaling,” the average of the *p* values of pathways “Toll-like receptor signaling,” “death receptor signaling,” “TNFR1 signaling,” “TNFR2 signaling” and “NF-κB signaling” was calculated. For each treatment, the average magnitude of the log2-fold change values of the genes responsible for the significance of the oxidative stress and inflammatory pathways was calculated and displayed as an arrow vector above the −log10 *p* value bars of the bar graph. The number of genes responsible for the significance of the individual pathways is color-coded from blue (low number of genes) to pink (high number of genes).

### Generation of GFP-tagged cell lines

HepG2 cells stably expressing human GFP-p65 as described in (Fredriksson et al. 2011). Mouse sulfiredoxin (Srxn1) was tagged with GFP at the C-terminus using BAC recombineering (Hendriks et al. 2012) and stably introduced into HepG2 cells by transfection and 500 μg/mL G-418 selection.

### RNA interference

siRNAs against human NFE2L2 (Nrf2) and KEAP1 were acquired from Dharmacon (ThermoFisher Scientific) as siGENOME SMARTpool reagents, as well as in the form of four individual siRNAs. HepG2 cells were transiently transfected with the siRNAs (50nM) using INTERFERin (Polyplus) as described previously (Fredriksson et al. 2011).

### Western blotting

Samples were collected by direct cell lysis (including pelleted apoptotic cells) in 1 × sample buffer supplemented with 5 % v/v β-mercaptoethanol and heat-denatured at 95 °C for 10 min. The separated proteins were blotted onto PVDF membranes before antibody incubation in 1 % BSA in TBS–Tween 20. The following antibodies were used: mouse-anti-GFP (Roche); rabbit-anti-IκBα (Cell Signaling); rabbit-anti-Nrf2 (H300, Santa-Cruz); mouse-anti-Cleaved Caspase-8 (Cell Signaling); rabbit-anti-PARP (Cell Signaling); mouse-anti-Tubulin (Sigma); mouse-anti-actin (Santa-Cruz).

### Microscopy

Real-time cell death induction was determined by monitoring the accumulation of Annexin-V-Alexa633-labeled cells over a 24-h time period (Puigvert et al. 2010). For this, transmission and Alexa633 images of the same area with cells were taken automatically every 30 min using a BD Pathway™ 855 bioimager with CCD camera and a 10x objective with an image resolution of 608 × 456 (binning 2).

Accumulation of Srxn1-GFP or nuclear oscillation of GFP-p65 was monitored using a Nikon Eclipse Ti confocal microscope (lasers: 488 and 408 nm), equipped with an automated stage, Nikon 20x Dry PlanApo VC NA 0.75 objective and perfect focus system. Images were acquired at 512 × 512 pixels. Prior to imaging at >20× magnification, HepG2 cells were loaded for 45 min with 100 ng/mL Hoechst_33342_ to visualize the nuclei, upon which the Hoechst-containing medium was washed away to avoid Hoechst phototoxicity (Purschke et al. 2010). Srxn1-GFP cells were imaged every 30 min across a 24-h time span, and GFP-p65 cells every 6 min for 6 h.

### Image quantification

To quantify the total pixel area occupied by cells or the number of cells per field imaged, transmission images and Hoechst images, respectively, were analyzed using ImagePro 7.0 (Media Cybernetics). The accumulation of dead cells or the appearance of Srxn1-GFP-positive cells was quantified as the total number of pixels above background. The Annexin-V-positive pixel total was normalized for the total cell area. The number of adjacent fluorescent Srxn1-GFP pixels above background (with a minimum size of 45 pixels, which is about one-fourth of average cell size) was multiplied by the average density of those pixels as a measure for the GFP signal intensity increase and normalized for the amount of nuclei.

To quantify the nuclear translocation of GFP-p65, nuclei (Hoechst) masks are segmented and tracked in ImageJ to define the GFP-p65 nuclear intensity, followed by cytoplasm segmentation. The normalized nuclear/cytoplasmic intensity ratio for each cell is recorded and further analyzed for different oscillation features, also using ImageJ, including the number of translocations, time period of each individual peak, intensity of the peaks, delay between peaks, and nuclear entry and exit rates (Di et al. 2012).

### Statistics

All experiments are performed at least in triplicate. Error bars indicate standard error. Statistical comparisons were made using a one-way ANOVA. The following *p* values were considered significant: *p* < 0.05 (*); *p* < 0.01 (**); *p* < 0.001 (***).

## Results

### Enhanced Nrf2 activation is associated with suppression of endogenous NF-κB activity in PHH

**Fig. 1:**
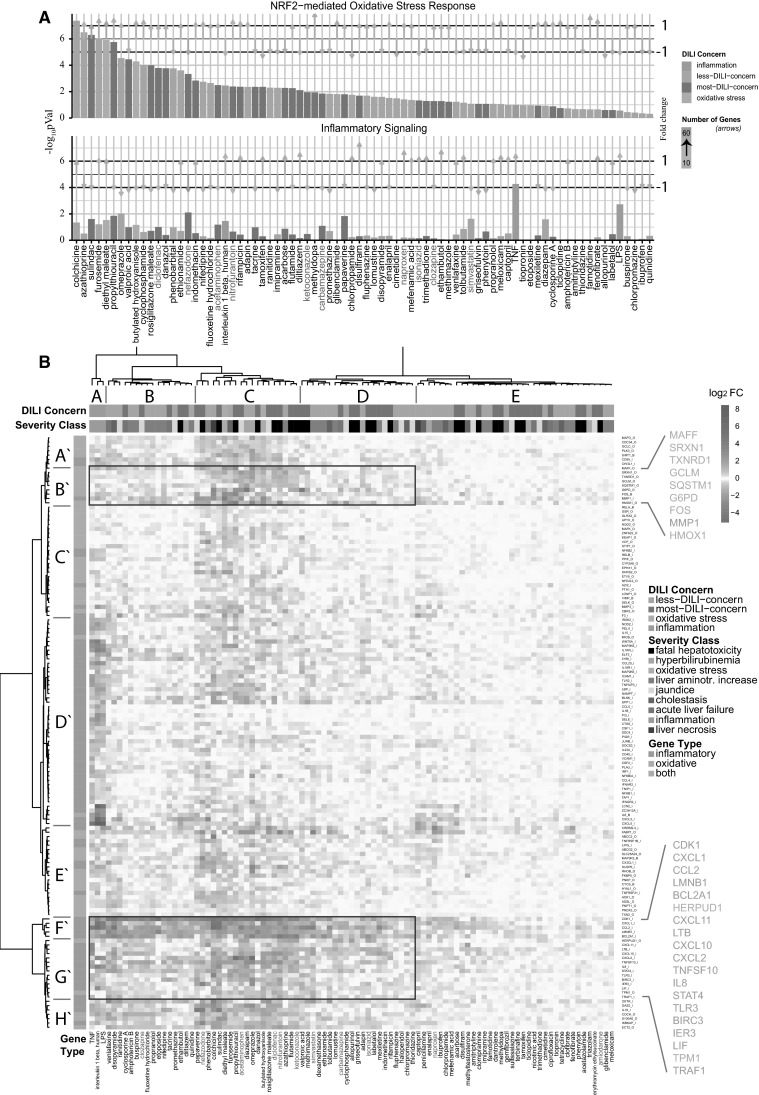
Gene expression analysis of 24-h highest concentration primary human hepatocyte subset of the TG-GATEs dataset. **a** Differentially expressed genes were analyzed with Ingenuity Pathway Analysis as described in detail in the “Materials and methods” section. In the top panel, the −log_10_ *p* values for the corresponding pathways are displayed for the Nrf2-mediated oxidative stress response. The *top panel* displays the mean of the *p* values for the inflammatory-related pathways. Compounds are ordered according to highest significance of the Nrf2-mediated oxidative stress response. The compound labels in *red* are the compounds chosen in this study. The *color of the bars* corresponds to DILI severity type or to the oxidative stress/inflammatory model compounds (model compound type). The length of the *arrows* corresponds to the mean fold change of the genes which are responsible for the significance of the corresponding pathways. The direction of the *arrow* corresponds to either mean up- or downregulation of these genes. The *color of the arrows* corresponds to the number of these genes ranging from 10 to 60 genes. **b** Unsupervised hierarchical clustering of all DILI compounds and a selected gene set as described in detail in the “Materials and methods” section. *Blue* corresponds to downregulated genes, and orange, to upregulated genes; the brightness corresponds to the magnitude of the fold changes. The top color-coded bar corresponds to the DILI concern or model compound type. The second top color-coded bar corresponds to the severity class or model compound type. The left color-coded bar corresponds to the gene type—either inflammatory genes, oxidative genes or both. Important clusters on gene level are represented from *A*′ to *H*′, and important compound-level clusters with *A*–*E* for easy reference from the text. Compounds used in this study are *color-coded in red* (color figure online)

The Japanese Toxicogenomics Project has generated the Open TG-GATEs data repository of gene expression profiles in PHH upon exposure to 157 compounds, of which many are DILI-related, at 1–3 different concentrations and 1–3 time points (2, 8 and 24 h), including a few pro-inflammatory cytokines, TNFα, IL1β and LPS (Uehara et al. 2010). We focused on the NF-κB and Nrf2 signaling-related gene sets extracted from several key databases as described in detail in the “Materials and methods” section. Ingenuity Pathway Analysis (IPA) for oxidative stress and inflammatory signaling was performed for all DILI compounds in the dataset. Typically, a significant modulation of these pathways was observed. A major modulation of the “Nrf2-mediated oxidative stress response” overall related to upregulation of genes linked to this pathway. Interestingly, DILI compounds that showed a strong oxidative stress response also showed a modulation of “inflammatory signaling” related to NF-κB activity (26 compounds, *p* < 0.05) although this was typically associated with downregulation of genes (Fig. 1a). This effect was strongest after 24-h treatment, although a similar association was already observed at 8-h treatment (Supplementary Fig. 2A).

The above observation indicated an opposite direction of Nrf2-mediated signaling versus NF-κB-related inflammatory signaling by DILI compounds in PHH. Indeed, Nrf2 can negatively affect NF-κB activity (Liu et al. 2008; Yu et al. 2011). Therefore, we next performed a more detailed hierarchical clustering analysis of the altered gene expression induced by all DILI compounds associated with both signaling pathways. As a first step, based on different annotation databases, we systematically selected a set of Nrf2 signaling-related genes as well as a set of inflammatory signaling-related genes. To determine which genes are responsive to oxidative stress and inflammatory stimuli in PHH, we included a stringent filtering procedure based on the exposures of PHH in the TG-GATEs data to DEM and BHA for Nrf2 signaling, and TNFα, IL-1β and LPS for inflammatory signaling. We then extracted the differential expression levels for all DILI compounds for the selected 55 and 82 genes related to Nrf2 signaling and inflammatory signaling, respectively. Using an unsupervised hierarchical clustering for all genes and DILI compounds at 24 h, we could clearly distinguish Nrf2 clusters (A′, B′, C′ and E′) and NF-κB gene clusters (D′, F′ and G′) (Fig. 1b). Moreover, cytokines and LPS (cluster A) clearly induced a different response compared to all DILI compounds (clusters B–E). DILI compound cluster C gave the strongest overall response at the level of both Nrf2 target gene activation and inflammation signaling target gene downregulation; this cluster was slightly enriched in compounds that demonstrate “fatal hepatotoxicity”. These effects were not as prominent at 8-h treatment conditions (Supplementary Fig. 2B).

**Fig. 2:**
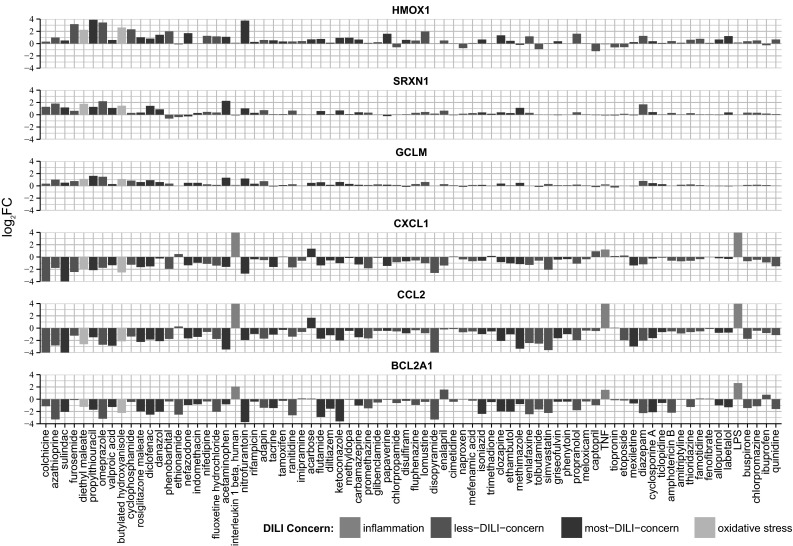
Fold changes of example genes from the two prominent clusters from the unsupervised hierarchical cluster analysis. Oxidative stress genes HMOX, SRXN1, GCLM (*blue*) from cluster *B′* from Fig. 1b and inflammatory genes CXCL1, CCL2, BCL2A1 (*purple*) from clusters *F*′ and *G*′. *Color codes* are as in Fig. 1 (color figure online)

Within the hierarchical cluster analysis, two strong gene clusters were prominent in their response to DILI compounds: a first cluster (cluster B′) with Nrf2 target genes that were mostly upregulated by DILI compounds but hardly affected by cytokines, including Maff, Srxn1, Txnrd1, GCLM, SQSTM1, G6PD, FOS, MMP1 and HMOX1, mostly prototypical Nrf2 target genes (see Fig. 2 for examples), and a second cluster (clusters F′ and G′) with inflammatory genes that were strongly upregulated by the cytokines and LPS, but were strongly downregulated by the same DILI compounds that caused upregulation of Nrf2 targets, which included CXCL1, CCL2, BCL2A1, CXCL11, CXCL2 (see Fig. 2 for examples). To determine the correlation with the DILI severity, we performed a similar cluster analysis for only severe DILI compounds and non-severe DILI compounds based on the FDA drug labeling classification (Chen et al. 2011) (Supplementary Figs. 3 and 4). Severe DILI compounds mostly mimicked the overall DILI hierarchical cluster analysis showing the strongest inverse relationship between Nrf2 activity and NF-κB suppression and included DCF, sulindac, ketoconazole (KTZ) and acetaminophen (APAP).

Altogether, these findings indicate a strong correlation between the ability of DILI compounds to induce an adaptive Nrf2 response and the suppression of NF-κB activity.

### A BAC-Srxn1-GFP HepG2 cell line reports xenobiotic-mediated Nrf2 activation

**Fig. 3:**
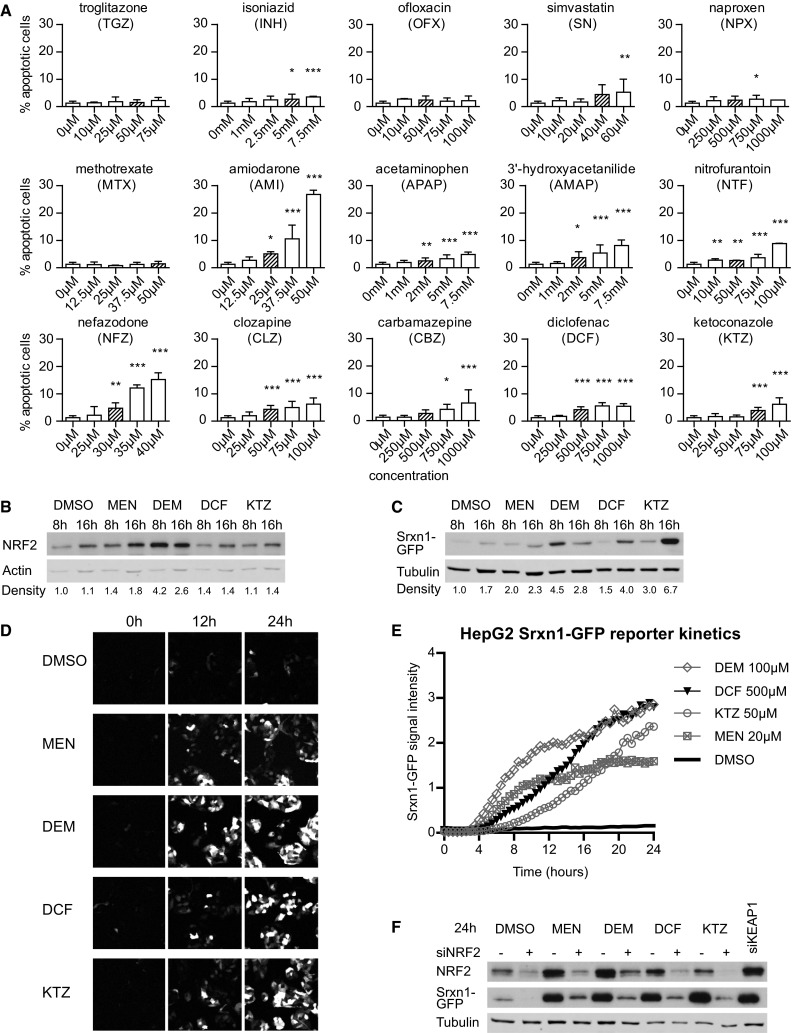
Srxn1-GFP BAC HepG2 reporter cell line is dependent on Nrf2/KEAP1 signaling. **a** Cell injury assay using Annexin-V-Alexa-633 staining after 24-h exposure to our compound set. **b** Western blot of Nrf2 expression in HepG2 cells exposed for 8 or 16 h to MEN, di-ethyl maleate (DEM), diclofenac (DCF) or KTZ. Density quantification is relative to actin levels, normalized to DMSO. **c** Western blot of GFP expression in HepG2 Srxn1-GFP cells as in **b**. Density quantification below is relative to tubulin levels. **d** Stills of time-lapse imaging of HepG2 Srxn1-GFP cells exposed to Nrf2 inducers. **e** Quantification of the Srxn1-GFP reporter response kinetics. **f** siRNA-mediated knockdown of Nrf2 (+siNrf2) or KEAP1 (siKEAP1) or mock treatment (−) in HepG2 Srxn1-GFP cells exposed to DMSO, MEN, DEM, DCF or KTZ for 24 h

The most prominent differences between NF-κB and Nrf2 responses in the PHH dataset were observed at high concentrations and at 24 h of drug exposure. Like all signaling events, the transcriptional activities of Nrf2 and NF-κB are dynamic in nature and may show differential activity over time. Therefore, we sought to monitor the activity of these two transcription factors in living cells using GFP-tagging technology allowing their dynamic analysis. As PHH dedifferentiate within 24 h in vitro when grown in 2D cultures (Boess et al. 2003) and are not amenable for stable expression of GFP reporter constructs, we chose the liver model cell line HepG2 to generate stable fluorescent reporters for both NF-κB and Nrf2 signaling. As a first step, to enable reliable quantitative measurements of the dynamic effect of drug exposure on Nrf2 activity using live-cell imaging, we generated a HepG2 reporter cell line based on bacterial artificial chromosome (BAC) recombineering (Poser et al. 2008) of the Nrf2 target gene sulfiredoxin (Srxn1) (Hendriks et al. 2012), which was part of the predictive DILI cluster. We tagged the Srxn1 gene with GFP at its C-terminus and established a stably expressing HepG2 Srxn1-GFP cell line under control of its own entire promoter region. To monitor for its functionality as an Nrf2 reporter, we exposed the HepG2 cells to MEN (20 μM) and DEM (100 μM) as proto-typical model activators of Nrf2, as well as DCF and KTZ, of which the PHH data revealed their capacity to strongly activate an Nrf2 response. DEM, MEN, DCF and KTZ all stabilized Nrf2 levels in our cells (Fig. 3b, c). Live-cell imaging by confocal microscopy followed by automated image quantification showed that the Srxn1-GFP reporter is activated with different kinetics by different compounds with MEN and DEM being fast inducers, likely related to their direct mode-of-action, and DCF and KTZ showing a delayed response, possibly related to bioactivation (Fig. 3b–e); this effect was directly related to the expression of the GFP-Srxn1 fusion product. Finally, to confirm that our Srxn1-GFP reporter is under direct control of the KEAP1/Nrf2 pathway, we transiently transfected the HepG2 Srxn1-GFP cells with siRNA oligos targeting Nrf2 or KEAP1. siRNA targeting Nrf2 prevented the stabilization of Nrf2 and consequently inhibited the Srxn1-GFP induction for all compounds. In contrast, as expected, KEAP1 knockdown itself stimulated Srxn1-GFP expression (Fig. 3f). These data show that the Srxn1-GFP signal intensity depends on the functional KEAP1/Nrf2 pathway.

### Drug-induced cell death of human HepG2 cells

Next, we selected a set of DILI compounds for further characterization. Since the opposite regulation of Nrf2 versus NF-κB by DILI compounds was largely seen for severe DILI compounds that often require bioactivation, we selected a small panel of compounds that was contained within the TG-GATEs dataset [APAP, carbamazepine (CBZ), clozapine (CLZ), DCF, KTZ, nitrofurantoin (NTF) and nefazadone (NFZ)] as well as some DILI compounds that do not require bioactivation and do not activate the Nrf2 pathway much in PHH [amiodarone (AMI), NPX and simvastatin (SN)]; we further complemented our compound set with a few additional drugs that fit in these categories but were not included in the TG-GATEs [ofloxacin (OFX), isoniazid (INH), methotrexate (MTX), 3′-hydroxyacetanilide (AMAP) and troglitazone (TGZ)] (Supplementary Table 2). We first tested whether these compounds induced sufficient cell injury that resulted in cell death at similar concentrations as used for the PHH dataset (Fig. 3a). Based on automated live-cell imaging of Annexin-V-positive cells, we identified concentration-dependent HepG2 cell death for AMI, APAP, AMAP, CBZ, CLZ, DCF, KTZ, NFZ, NTF and SN. Little cell death was observed for INH, MTX, NPX, OFX and TGZ. For further experiments, we continued with a mildly cytotoxic concentration (<10 % apoptosis onset) for each compound (indicated in Supplementary Fig. 5) to establish the effect on Nrf2 activation, NF-κB signaling and the cytotoxic interaction between DILI compounds and the pro-inflammatory cytokine TNFα.

### DILI compounds activate the Nrf2 stress response independent of TNFR activation

**Fig. 4:**
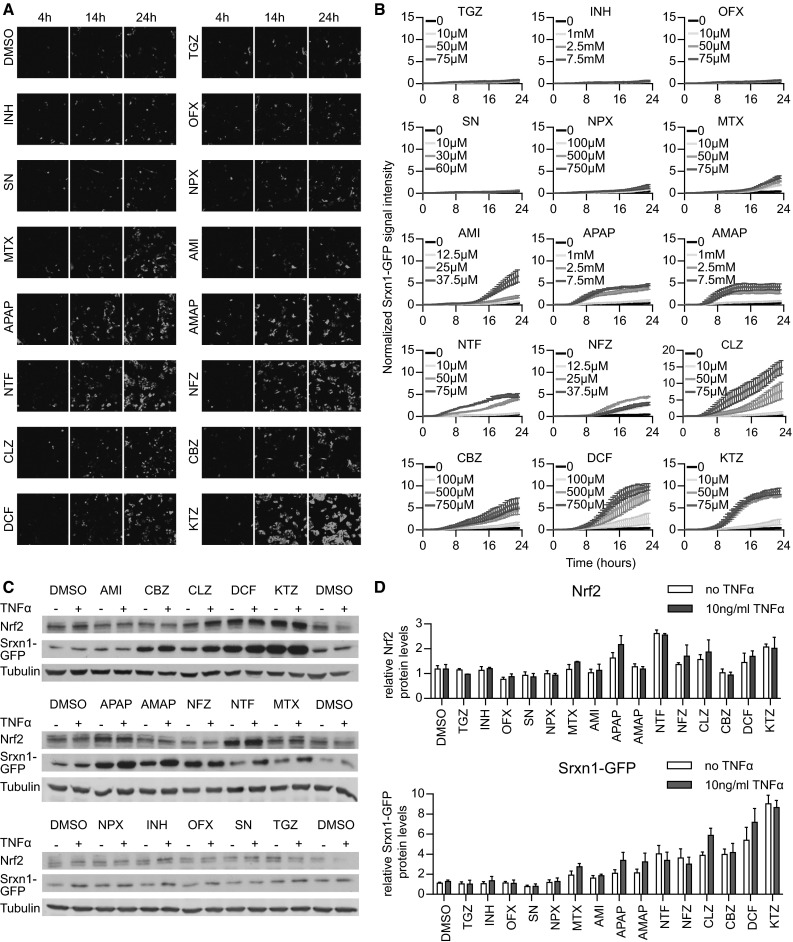
Drug exposure induces dynamically divergent Nrf2 responses. **a** Stills of confocal live-cell imaging in HepG2 Srxn1-GFP cells upon drug exposure (shown are 4, 14 and 24 h). **b** Quantification of the Srxn1-GFP signal appearing upon exposure to increasing drug doses (averages shown of four independent replicates). **c** Western blots for Nrf2 and GFP expression after 24-h drug exposure in HepG2 Srxn1-GFP cells, either with or without co-exposure to 10 ng/mL TNFα. **d** Quantification of the Nrf2 and Srxn1-GFP protein levels, 24 h after drug ±TNFα exposure (averages of three replicates)

The PHH dataset predicted that APAP, CBZ, CLZ, DCF, KTZ and NTF potently activate the Nrf2 response; that INH, NFZ and NPX mildly induce Nrf2; and that AMI and SN weakly activate it (Supplementary Fig. 6). Using live-cell imaging, we tested whether these same drugs activated the Srxn1-GFP response in HepG2 cells (Fig. 4a, b). We observed that APAP induced the oxidative stress reporter as soon as 4 h after compound exposure, which is remarkable considering the low CYP2E1 levels in HepG2 cells; however, this does indicate that the HepG2 is sensitive to oxidative stress adaptation signaling. Possibly, APAP induces oxidative stress through other means than CYP2E1-mediated bioactivation, possibly involving direct modulation of the mitochondrial respiratory chain. NTF, DCF, KTZ, CLZ, CBZ and NFZ strongly induced the Srxn1-GFP reporter as early as 8 h after compound exposure. AMI, MTX and NPX showed weak Srxn1-GFP induction with delayed kinetics, around 16 h after compound exposure. INH, OFX, SN and TGZ did not lead to oxidative stress induction within the 24-h imaging period in our cell system. These findings indicate that the PHH results on the Nrf2 pathway activation correlate well with the HepG2 Srxn1-GFP reporter cell observations.

TNFα promotes NF-κB target gene activation through binding to TNFRSF1A. TNFα binding to its receptor has been suggested to promote Nrf2 activation (Rushworth et al. 2011), while the PHH dataset predicted no effect of TNFα on Nrf2 responses. To confirm this, we tested whether drug exposure in combination with 10 ng/mL TNFα influenced the drug-induced Nrf2 response (Fig. 4c, d). We observed neither a significant rise nor a decrease in Nrf2 stabilization or Srxn1-GFP expression at 24 h when the HepG2 Srxn1-GFP cells were exposed to TNFα alone or in combination with an 8-h drug pre-exposure. This suggests that TNFα-mediated NF-κB signaling does not influence Nrf2 target gene activation caused by deleterious DILI compounds.

### DILI compounds cause a perturbation of NF-κB signaling

**Fig. 5:**
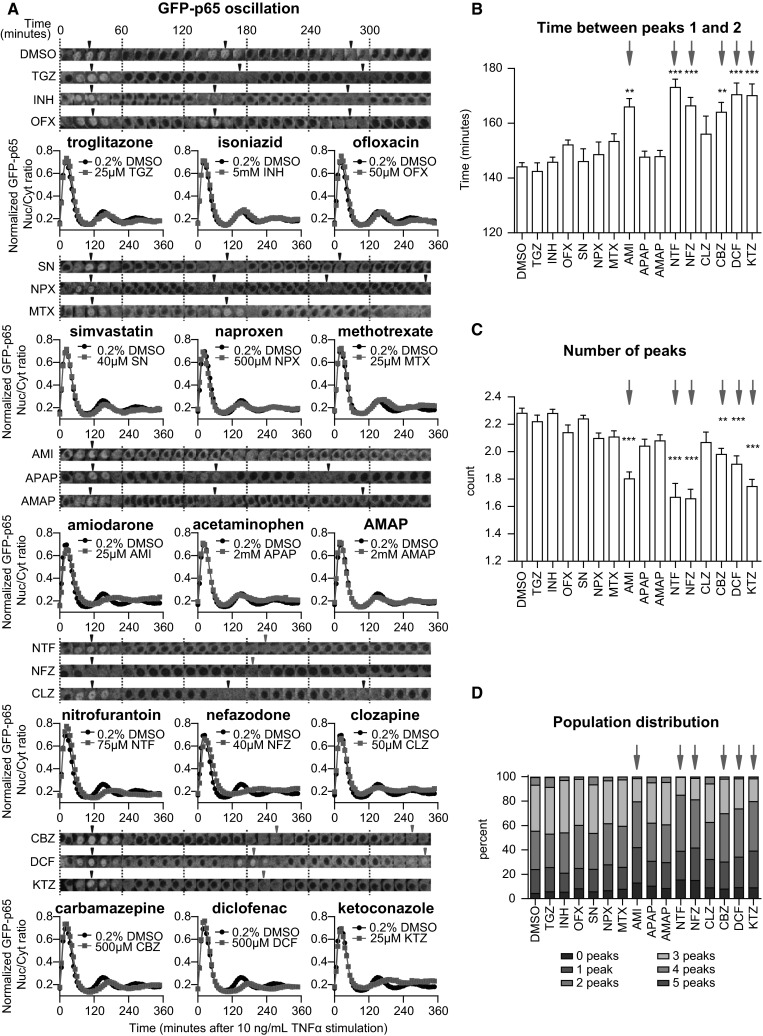
DILI compounds affect the TNFα-mediated nuclear translocation response of NF-κB. **a** Time-lapse images of one cell that illustrates NF-κB oscillation upon 10 ng/mL TNFα stimulation after an 8-h drug pre-incubation period. *Arrowheads* point at the local nuclear translocation maxima (“peaks”). Quantified average of the GFP-p65 nuclear/cytoplasmic intensity ratio (average of three experiments, totaling 800–1200 cells), normalized between 0 and 1 to focus on the appearance of the nuclear translocation maxima. **b** Analysis of the NF-κB response: time between peaks 1 and 2. **c** Analysis of the NF-κB response: assessment of the number of peaks. **d** Distribution of the TNFα-stimulated, drug pre-exposed cell population, classified for showing 0–5 peaks within the 6-h imaging period

To test whether Nrf2 activation by DILI compounds is associated with modulation of NF-κB signaling, we made use of a previously established HepG2 cell line expressing GFP-tagged p65/RelA, a subunit of the dimeric transcription factor NF-κB (Fredriksson et al. 2011). As reported (Fredriksson et al. 2011), an 8-h DCF pre-exposure delays the second translocation event (peaking 26 min later than vehicle pre-incubated cells) (Fig. 5a). Also NTF (+29 min), KTZ (+26 min), AMI (+22 min), NFZ (+22 min) and CBZ (+20 min) delayed the oscillation to a similar extent as DCF. Pre-treatment with CLZ and MTX only weakly perturbed the appearance of the second translocation response with a delay of 12 and 9 min, respectively. Neither AMAP, APAP, INH, OFX, SN nor TGZ significantly influenced the translocation maximum of the second nuclear translocation event.

Our live-cell imaging approach allowed detailed cell population-based quantitative analysis of the translocation response to extract various relevant parameters that describe the NF-κB oscillation pattern invoked by TNFα at the single cell as well as the cell population level (Di et al. 2012). This analysis revealed that pre-treatment with AMI, CBZ, DCF, KTZ, NFZ or NTF significantly delayed the time between the first and second NF-κB nuclear translocation maxima that normally occur at 30 and 150 min after TNFα exposure, respectively (Fig. 5b). This effect limits the average number of translocation events observed within the 6-h imaging window (Fig. 5c). Importantly, by evaluating on average ~1000 cells per condition, we identified that AMI, CBZ, DCF, KTZ, NFZ and NTF induced a sharp decrease in the percentage of cells that undergo three or more NF-κB nuclear translocation events (Fig. 5d). Together, the results indicate that various DILI compounds affect the TNFα-induced NF-κB activation response by modulating its nuclear translocation dynamics. For the compounds with this delayed translocation event, the NF-κB target genes are downregulated (Fig. 1a) and all compounds except AMI fall within inhibited NF-κB/activated Nrf2 signaling clusters (clusters B–C, and CBZ cluster D, Fig. 1b), suggesting that the delayed translocation could be indicative of lower NF-κB target gene expression.

### The inhibitory effect of Nrf2 activity on NF-κB signaling promotes the pro-apoptotic role of TNFα in drug-exposed HepG2 cells

**Fig. 6:**
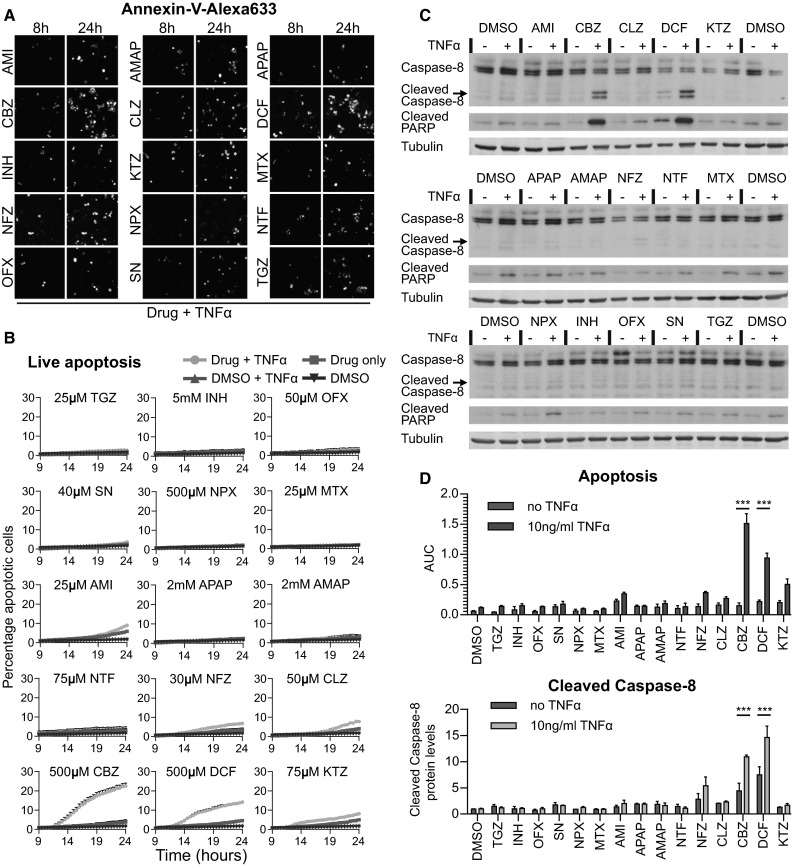
Adverse DILI compound and TNFα synergy for the onset of cell death. **a** Still images of time-lapse movies of HepG2 cells exposed to the drugs in the co-presence of Annexin-V-Alexa-633, taken at 8 h (before 10 ng/mL TNFα addition) and at 24 h (16 h TNFα). **b** Quantification of the percentage dead cells appearing upon drug only exposure, or in combination with TNFα. Average of 3–6 experiments. **c** Western blot for cleaved caspase-8 and the caspase substrate PARP, induced by 24-h drug alone or drug–TNFα co-treatment. **d** Comparison of the quantified percentage of dead cells 24 h after drug (+TNFα) exposure: the appearance of dead cells in live-cell imaging as area under the curve (AUC) (as in **b**) and quantification of cleaved caspase-8 protein levels (relative density as in **c**, average of three experiments)

TNFα-mediated signaling seems important in DILI (Cosgrove et al. 2009; Steuerwald et al. 2013). While TNFα-receptor-mediated NF-κB signaling may provide survival signaling through the upregulation of anti-apoptosis genes such as the anti-apoptotic Bcl-2 family member A1 (BCL2A1), activation of the TNFR may in parallel initiate activation of caspase-8 and therefore switch on apoptosis (Hsu et al. 1996). Since DILI compounds did affect the NF-κB signaling and therefore possibly suppressed survival signaling, we next investigated whether DILI compounds would also predispose to the onset of TNFα-mediated apoptosis. To address this issue, we monitored the rate of HepG2 cell apoptosis by live-cell imaging with Annexin-V-Alexa633 after 8-h drug pre-exposure and tested whether TNFα co-exposure enhanced the apoptotic response at 24 h. TNFα enhanced the apoptosis induction upon CBZ and DCF exposure by 18.6 and 9.7 %, respectively. A smaller increase of 3–4 % in cell death upon TNFα co-stimulation was found for KTZ, AMI, NFZ and CLZ (Fig. 6a, b). Since TNFα-mediated death signaling acts through caspase-8 activation, we anticipated that the synergy for the onset of apoptosis would also be associated with enhanced caspase-8 cleavage. Caspase-8 was markedly increased by TNFα combined with CBZ and DCF, yet for other DILI compounds tested, such a caspase-8 activation was not observed, as was expected based on the limited onset of apoptosis (Fig. 6c, d). The enhanced caspase-8 cleavage was associated with cleavage of PARP, a well-established caspase substrate which serves as a pivotal marker of onset of apoptosis. This indicates that primarily under CBZ and DCF pre-treatment conditions, co-treatment with TNFα turns on apoptosis.

## Discussion

Here, we focused on the interplay of two pivotal cellular stress response signaling pathways in DILI: TNFα-mediated NF-κB signaling and chemical stress-induced Nrf2 activation. Extensive transcriptomics data from primary human hepatocyte revealed that the Nrf2 transcriptional program is activated by a majority of different DILI compounds, in particular those that are associated with severe DILI. This strong Nrf2 activation correlates with a major downregulation of genes that are under the direct control of NF-κB. We successfully transferred this inverse relationship between Nrf2 activation and NF-κB signaling into a panel of GFP-reporter-based high-content imaging assays, which now allows the high-throughput assessment of their dynamic activation (Wink et al. 2014). Using live-cell imaging, we established the time profiles of the activation of these transcription factors and established that various DILI compounds activate Nrf2 activity as well as negatively modulate the NF-κB nuclear oscillation response induced by TNFα. Although no cause and effect relationship between these two signaling pathways has been proved in our study, our data do support an overall working model whereby DILI compounds that strongly affect the Nrf2 response as well as modulate the NF-κB oscillatory response (either directly or indirectly) act in synergy with TNFα to cause a cytotoxic response. An integrated automated high-throughput microscopy-based platform that simultaneously measures drug-induced Nrf2 activation, TNFα-induced NF-κB activation and cytotoxicity will likely contribute to the exclusion or de-prioritization of novel drug entities for further development.

Our data indicate a differential regulation of Nrf2 and NF-κB signaling pathways in PHH. From the Japanese Toxicogenomics Project, a total of 90 DILI compounds have been evaluated. While several DILI compounds caused a strong modulation of most Nrf2 and NF-κB target genes, e.g., NTF, DCF and KTZ, the effect of AMI was only modest. Despite the fact that HepG2 cells are notorious for their low level expression of CYP enzymes (Westerink and Schoonen 2007), an enhanced formation of reactive intermediates during drug metabolism may be causative for the activation of the Nrf2 response. However, we cannot exclude the role of other stress response pathways that are intricately linked to the modulation of the Nrf2 response and by themselves are activated by chemical-induced cell injury, including the perturbation of the mitochondria, the endoplasmic reticulum (ER) and the autophagosomes which may result in a secondary source of ROS that may initiate an adaptive Nrf2 response (Sano and Reed 2013). Although the role of these other programs will require further mechanistic investigations, our previous investigations demonstrate that suppression of the Nrf2 adaptive stress response strongly sensitizes cells toward a synergistic toxicity with TNFα, indicating that enhanced oxidative stress predisposes for TNFα sensitization (Fredriksson et al. 2014).

The PHH transcriptomics data indicated that many DILI compounds themselves suppress the activity of NF-κB target genes. In addition, our imaging data indicate that various DILI compounds suppress the NF-κB oscillatory response. Together, this suggests that also under control situations, the overall nuclear localization of NF-κB may be limited, thereby precluding the activation of NF-κB target genes. Alternatively, a limited activation of NF-κB by DILI compounds possibly influences the expression of modulators that act as feedback suppressors of NF-κB activity, such as IkBα/NFKBIA or A20/TNFAIP3 (Hutti et al. 2007). Indeed, NF-κB signals through an auto-regulatory negative feedback mechanism that essentially desensitizes a cell for a limited time period against re-activation of the response by an active NF-κB-inducing kinase complex (IKK) (Hinz and Scheidereit 2014). Although drug exposure alone may elicit NF-κB oscillations, this does not limit the primary nuclear translocation event upon TNFα exposure, only the subsequent nuclear translocation events. The later oscillations are less intense and less synchronized due to induction of a second negative feedback regulator, A20. Interestingly, several, but not all, DILI compounds affect the expression of IkBα and A20 in PHH, which often occur in parallel, supporting a similar mechanism of activation (see Supplementary Fig. 7). We therefore turned to our GFP-p65 reporter and tested whether the test drugs can induce NF-κB oscillations on their own. In line with this, we found that DCF, CBZ, NFZ, CLZ and KTZ induced a limited NF-κB transition in 2–6 % of a given cell population within the first 2 h after exposure which was not apparently different from control conditions (Supplementary Fig. 8). This suggests that drug pre-exposure does not directly change the initial balance of NF-κB and its cytoplasmic inhibitor, IκBα, but rather may influence the transcriptional and translational responses required for normal execution of the timing of the NF-κB response after the first nuclear translocation event.

The rationale for the choice of drugs was to investigate whether our live-cell imaging systems were able to discriminate between drugs that are often linked to DILI (TGZ, AMI, INH, KTZ, NFZ, MTX, NTF, CBZ and DCF) and relatively safe drugs (NPX, SN, OFX and CLZ). We have focused on NF-κB signaling, Nrf2 activation and cell death induction, and a summary of the different responses is provided in Table 1. As APAP and AMAP induce hepatocellular death through necrosis at high levels of drug concentrations (an EC_50_ in PHH of ~25 mM), and not apoptosis, these are considered as relatively safe drugs (Hadi et al. 2013). Based on our results, NPX, SN and OFX are safe (no massive cell death induction, no gross effect on Nrf2 or NF-κB signaling), but CLZ should be re-evaluated: Its profile of strong Srxn1-GFP induction, NF-κB delay and slightly higher cell death induced by TNFα co-exposure shows more resemblance to drugs that are more often associated with DILI, such as DCF, CBZ, KTZ, NFZ and NTF.

Our assays have not been able to pick up any mechanistic signs for toxicity for INH and TGZ, two typical idiosyncratic DILI-related drugs (Table 1). The hepatotoxic effect of these two drugs, however, could partly depend on their inhibitory effect on bile acid transport (Cheng et al. 2013; Foster et al. 2012), which might only emerge from advanced (3D) hepatocyte culture models (Malinen et al. 2012). Moreover, lack of strong bioactivation capacity in HepG2 cells could also be a reason why we could not observe any effect for these compounds.

In conclusion, we demonstrate an association between Nrf2 signaling and NF-κB responses in two distinct liver models: PHH and HepG2. Using the live-cell imaging of our GFP-based reporter models for Nrf2 and NF-κB signaling, we established the inverse relationship between these signaling pathways in relation to DILI compound and TNFα-mediated synergistic toxicity. This was only feasible by assessing the quantitative dynamics of the NF-κB responses, underscoring the integration of live-cell imaging of stress response pathways in mechanistic studies in relation to DILI assessment.

## Electronic supplementary material

Below is the link to the electronic supplementary material.

Supplementary material 1 (DOCX 31 kb)

Supplementary material 2 (PDF 62 kb)

Supplementary material 3 (PDF 65 kb)

Supplementary material 4 (EPS 2381 kb)

Supplementary material 5 (EPS 4142 kb)

Supplementary material 6 (EPS 1995 kb)

Supplementary material 7 (EPS 2134 kb)

Supplementary material 8 (EPS 1513 kb)

Supplementary material 9 (EPS 839 kb)

Supplementary material 10 (EPS 2021 kb)

Supplementary material 11 (EPS 1980 kb)

## Abbreviations

BHA: Butylated hydroxyanisole
DILI: Drug-induced liver injury
PHH: Primary human hepatocytes
siRNA: Small interfering RNA
ROS: Reactive oxygen species
APAP: Acetaminophen/paracetamol
AMAP: 3′-Hydroxyacetanilide
AMI: Amiodarone
CBZ: Carbamazepine
CLZ: Clozapine
DCF: Diclofenac
DEM: Di-ethyl maleate
INH: Isoniazid
KTZ: Ketoconazole
MEN: Menadione
MTX: Methotrexate
NFZ: Nefazodone
NPX: Naproxen
NTF: Nitrofurantoin
OFX: Ofloxacin
SN: Simvastatin
TGZ: Troglitazone
