# Supplementary material for: Activation of the Nrf2 response by intrinsic hepatotoxic drugs correlates with suppression of NF-κB activation and sensitizes toward TNFα-induced cytotoxicity
Source: Arch Toxicol. 2015 May 31;90:1163–79. doi: 10.1007/s00204-015-1536-3 (PMC4830895; doi:10.1007/s00204-015-1536-3)
Supplement: Supplementary file 1 — Supplementary material 1 (DOCX 31 kb) [file 204_2015_1536_MOESM1_ESM.docx]

**Supplementary Materials**

**Supplementary Table 1: TG-GATES compounds and DILI annotations.**

1: steatosis, 2: cholestasis, 3: liver aminotransferases increase, 4: hyperbilirubinemia, 5: Jaundice, 6: liver necrosis, 7: acute liver failure, 8: fatal hepatoxicity

**Supplementary Table 2: Drugs used in this study and their reported adverse effects on the liver.**

**Supplementary Fig. 1: Flow diagram of TG-GATEs informatics analysis.**

**Supplementary Fig. 2: Gene expression analysis of 8 hours highest concentration primary human hepatocyte subset of the TG-GATES dataset.**

(A) Differentially expressed genes were analyzed with Ingenuity Pathway Analysis as described in detail in the material and methods section. In the top panel the –log^10^ p-values for the corresponding pathways are displayed for the Nrf2-mediated oxidative stress response. The top panel displays the mean of the p-values for the inflammatory related pathways. Compounds are ordered according to highest significance of the Nrf2-mediated oxidative stress response. The compound labels in red are the compounds chosen in this study. The color of the bars correspond to DILI-severity type or to the oxidative stress/ inflammatory model-compounds (model-compound type). The length of the arrows correspond to the mean fold change of the genes which are responsible for the significance of the corresponding pathways. The direction of the arrow corresponds to either mean up- or down-regulation of these genes. The color of the arrows corresponds to the number of these genes ranging from 10 to 50 genes. (B) Unsupervised hierarchical clustering of all DILI compounds and a selected gene set as described in detail in the material and methods section. Blue corresponds to down- regulated genes and orange to up-regulated genes, the brightness corresponds to the magnitude of the fold changes. The top color-coded bar corresponds to the DILI-Concern or model-compound type. The second top color-coded bar corresponds to the Severity Class or model-compound type. The left color-coded bar corresponds to the gene type- either inflammatory genes, oxidative genes or both. Compounds used in this study are color-coded in red.

**Supplementary Fig. 3: Gene expression analysis of Most DILI-concern 24 hours highest concentration primary human hepatocyte subset of the TG-GATES dataset.**

Unsupervised hierarchical clustering of most DILI-concern compounds and a selected gene set as described in detail in the material and methods section. Blue corresponds to down- regulated genes and orange to up-regulated genes, the brightness corresponds to the magnitude of the fold changes. The top color-coded bar corresponds to the DILI-Concern or model-compound type. The left color-coded bar corresponds to the gene type- either inflammatory genes, oxidative genes or both.

**Supplementary Fig. 4: Gene expression analysis of Less DILI-concern 24 hours highest concentration primary human hepatocyte subset of the TG-GATES dataset.**

Unsupervised hierarchical clustering of less DILI-concern compounds and a selected gene set as described in detail in the material and methods section. Blue corresponds to down- regulated genes and orange to up-regulated genes, the brightness corresponds to the magnitude of the fold changes. The top color-coded bar corresponds to the DILI-Concern or model-compound type. The left color-coded bar corresponds to the gene type- either inflammatory genes, oxidative genes or both.

**Supplementary Fig. 5:** **Drug-induced cell death of HepG2 cells.**

Percentage of dead HepG2 cells at 24 hours after exposure to fifteen different drugs. Concentrations are indicated in μM, except for AMAP, APAP and isoniazid (INH): in mM. “0”: 0.2% (v/v) DMSO. Shaded bars: concentration used in subsequent assays.

**Supplementary Fig. 6: Time course fold change of SRXN1 for PHH highest concentration (TG-GATES).**

Time course SRXN1 transcript level in PHH cells from the TG-GATES dataset. Fold change is expressed as log_2_(FC) as compared to matched vehicle controls. The different concentrations are color coded as low (blue), green (Middle) and red (High).

**Supplementary Fig. 7: Fold change values for IκB­α and A20 for PHH highest concentration (TG-GATES).**

Fold change is expressed as log_2_(FC) as compared to matched controls. Compounds are color coded according to DILI concern: inflammatory model compound (purple), oxidative stress model compound (blue), less-DILI-concern (light orange), most-DILI-concern (dark orange). Compounds are sorted according to log^2^(FC) of A20 from low to high.

**Supplementary Fig. 8:** **Drug-induced GFP-p65 oscillation in HepG2 cells.**

Quantified average of the GFP-p65 nuclear/cytoplasmic intensity ratio, normalized between 0 and 1 to focus on the appearance of the nuclear translocation maxima after exposure to 0.2% (v/v) DMSO (solvent control), 50µM CLZ, 500µM DCF, 500µM CBZ, 75µM KTZ and 40µM NFZ. Black line: average population response; Green line: average response of cells that show NF-κB nuclear translocation within the first 2 hours of compound addition.
