# Supplementary material for: Activation of the Nrf2 response by intrinsic hepatotoxic drugs correlates with suppression of NF-κB activation and sensitizes toward TNFα-induced cytotoxicity
Source: Arch Toxicol. 2015 May 31;90:1163–79. doi: 10.1007/s00204-015-1536-3 (PMC4830895; doi:10.1007/s00204-015-1536-3)
Supplement: Supplementary file 2 — Supplementary material 2 (PDF 62 kb) [file 204_2015_1536_MOESM2_ESM.pdf]

compound	conc.(μM)	Severity Class	DILI-Concern	DILI-Score
acetaminophen	5000	5	Most-DILI-Concern	DILI-Reports
isoniazid	10000	8	Most-DILI-Concern	Severe-DILI
phenobarbital	10000	3	Less-DILI-Concern	NA
valproic acid	5000	8	Most-DILI-Concern	Severe-DILI
clofibrate	300	3	Less-DILI-Concern	NA
rifampicin	70	8	Most-DILI-Concern	DILI-Reports
omeprazole	600	4	Less-DILI-Concern	NA
indomethacin	200	5	Most-DILI-Concern	High-Concern
chlorpromazine	20	2	Less-DILI-Concern	DILI-Reports
carbamazepine	300	7	Most-DILI-Concern	High-Concern
diclofenac	400	8	Most-DILI-Concern	High-Concern
nitrofurantoin	125	8	Most-DILI-Concern	High-Concern
diazepam	250	4	Less-DILI-Concern	NA
cyclophosphamide	2000	5	Less-DILI-Concern	NA
phenytoin	60	8	Most-DILI-Concern	NA
allopurinol	140	8	Most-DILI-Concern	NA
propylthiouracil	4000	8	Most-DILI-Concern	NA
gemfibrozil	100	3	Less-DILI-Concern	NA
amiodarone	7	8	Most-DILI-Concern	Severe-DILI
sulfasalazine	150	5	Most-DILI-Concern	NA
cimetidine	300	2	Less-DILI-Concern	NA
haloperidol	20	5	Less-DILI-Concern	NA
fluphenazine	20	3	Less-DILI-Concern	NA
thioridazine	15	5	Less-DILI-Concern	NA
adapin	75	4	Less-DILI-Concern	NA
labetalol	140	8	Most-DILI-Concern	Severe-DILI
methyltestosterone	20	2	Less-DILI-Concern	NA
glibenclamide	20	3	Less-DILI-Concern	DILI-Reports
griseofulvin	20	8	Most-DILI-Concern	NA
flutamide	50	8	Most-DILI-Concern	Severe-DILI
azathioprine	73	3	Less-DILI-Concern	High-Concern
ketoconazole	15	8	Most-DILI-Concern	Severe-DILI
tetracycline	25	2	Less-DILI-Concern	High-Concern
lomustine	120	3	Less-DILI-Concern	NA
ciprofloxacin	25	7	Most-DILI-Concern	NA
tamoxifen	25	8	Most-DILI-Concern	High-Concern
methyldopa	50	8	Most-DILI-Concern	High-Concern
methimazole	10000	8	Most-DILI-Concern	DILI-Reports
tacrine	80	7	Most-DILI-Concern	High-Concern
imipramine	15	3	Less-DILI-Concern	High-Concern
amitriptyline	15	5	Less-DILI-Concern	NA
ibuprofen	150	3	Less-DILI-Concern	DILI-Reports
naproxen	600	3	Less-DILI-Concern	High-Concern
quinidine	50	3	Less-DILI-Concern	NA
furosemide	2500	2	Less-DILI-Concern	DILI-Reports
fenofibrate	30	3	Less-DILI-Concern	NA
chlorpropamide	750	2	Less-DILI-Concern	DILI-Reports
nicotinic acid	10000	7	Most-DILI-Concern	NA
erythromycin ethylsuccinate	5	5	Most-DILI-Concern	NA
ethambutol	4000	8	Most-DILI-Concern	NA
mefenamic acid	150	3	Less-DILI-Concern	NA
famotidine	700	3	Less-DILI-Concern	DILI-Reports
ranitidine	4000	5	Less-DILI-Concern	High-Concern
nifedipine	150	3	Less-DILI-Concern	DILI-Reports
diltiazem	150	4	Most-DILI-Concern	NA
captopril	8000	6	Less-DILI-Concern	NA
enalapril	2000	4	Less-DILI-Concern	High-Concern
papaverine	60	5	Most-DILI-Concern	NA
penicillamine	10000	2	Less-DILI-Concern	NA
sulindac	3000	8	Most-DILI-Concern	High-Concern
disopyramide	3500	2	Less-DILI-Concern	NA
mexiletine	300	3	Most-DILI-Concern	NA

acetazolamide	600	8	Most-DILI-Concern	NA
disulfiram	60	8	Most-DILI-Concern	High-Concern
colchicine	4000	6	Less-DILI-Concern	NA
tolbutamide	2000	2	Less-DILI-Concern	DILI-Reports
acarbose	10000	8	Most-DILI-Concern	NA
simvastatin	30	3	Less-DILI-Concern	High-Concern
meloxicam	50	3	Less-DILI-Concern	NA
ethionamide	600	3	Less-DILI-Concern	NA
ticlopidine	20	4	Most-DILI-Concern	High-Concern
tiopronin	2000	3	Less-DILI-Concern	NA
promethazine	35	5	Less-DILI-Concern	NA
dantrolene	10	8	Most-DILI-Concern	Severe-DILI
clomipramine	10	8	Most-DILI-Concern	DILI-Reports
terbinafine	15	8	Most-DILI-Concern	High-Concern
danazol	35	8	Most-DILI-Concern	NA
etoposide	330	3	Less-DILI-Concern	NA
venlafaxine	1200	7	Less-DILI-Concern	DILI-Reports
clozapine	50	5	Most-DILI-Concern	High-Concern
buspirone	30	3	Less-DILI-Concern	Transaminitis
nefazodone	30	8	Most-DILI-Concern	Severe-DILI
triazolam	10	4	Less-DILI-Concern	NA
trimethadione	10000	5	Most-DILI-concern	NA
cyclosporine A	6	7	Most-DILI-Concern	NA
diethyl maleate	1500	oxidative stress	oxidative stress	NA
LPS	300	inflammation	inflammation	NA
TNF	50	inflammation	inflammation	NA
interleukin 1 beta, human	50	inflammation	inflammation	NA
butylated hydroxyanisole	200	oxidative stress	oxidative stress	NA
amphotericin B	2	3	Less-DILI-Concern	NA
fluoxetine hydrochloride	20	3	Less-DILI-Concern	DILI-Reports
dexamethasone	300	3	Less-DILI-Concern	Transaminitis
rosiglitazone maleate	300	5	Most-DILI-Concern	High-Concern
propranolol	100	3	Less-DILI-Concern	DILI-Reports
