# Supplementary material for: Activation of the Nrf2 response by intrinsic hepatotoxic drugs correlates with suppression of NF-κB activation and sensitizes toward TNFα-induced cytotoxicity
Source: Arch Toxicol. 2015 May 31;90:1163–79. doi: 10.1007/s00204-015-1536-3 (PMC4830895; doi:10.1007/s00204-015-1536-3)
Supplement: Supplementary file 3 — Supplementary material 3 (PDF 65 kb) [file 204_2015_1536_MOESM3_ESM.pdf]

Drug name	Abbreviation	Function	Type	Metabolizing enzymes	Adverse drug reactions in the liver	References
amiodarone	AMI	antiarrhythmic agent	positive control	CYP3A4; 1A2	ALT/AST elevations; cirrhosis; jaundice; hepatomegaly; hepatitis; phospholipidosis; steatohepatitis; cholestasis	[1, 2]
3'-hydroxyacetanilide	AMAP	regioisomer of paracetamol	negative control	CYP2E1	Safe in mice	[3, 4]
paracetamol / acetaminophen	APAP	analgesic and antipyretic	positive control	CYP2E1; 1A2; 2D6; 3A4	DRESS syndrome; acute liver failure; necrosis	[5-7]
carbamazepine	CBZ	antiepileptic drug	positive control	CYP3A4; 2C9; induces CYP3A4	SJS/TEN (Stevens-Johnson syndrome / toxic epidermal necrolysis); chronic hepatitis	[8-11]
clozapine	CLZ	antipsychotic drug	positive control	CYP3A4; 1A2; 2D6	ALT/AST elevations; hepatitis; jaundice; necrosis	[12-16]
diclofenac	DCF	NSAID	positive control	CYP3A4; 2C9 ; 2C8; UGT2B7	SJS; acute hepatitis; necrosis; autoimmune chronic liver injury	[17-19]
isoniazid	INH	anti-tuberculosis drug	positive control	CYP2E1; inhibits CYP2C9 and 3A4	ALT/AST elevation; acute hepatitis; chronic hepatitis; necrosis	[20-22]
ketoconazole	KTZ	antifungal antibiotic	positive control	CYP3A4; inhibits CYP3A4 and UGT2B7	acute hepatitis; cholestasis; necrosis	[23-25]
methotrexate	MTX	chemotherapeutic agent	positive control	aldehyde oxidase; CYP2E1	ALT/AST elevations; fibrosis; cirrhosis; chronic hepatitis; NASH	[26, 27]
nefazodone	NFZ	antidepressant	positive control	CYP3A4; inhibits CYP3A4	liver failure; jaundice; hepatitis; hepatocellular necrosis	[28, 29]
naproxen	NPX	NSAID	negative control	CYP2C9	ALT/AST elevations; cholestasis; acute hepatitis	[30]
nitrofurantoin	NTF	antibiotic against urinary tract infections	positive control	CYP1A	autoimmune hepatitis; chronic active hepatitis; necrosis	[31, 32]
ofloxacin	OFX	antibiotic	positive control	CYP1A2; 2C19	SJS/TEN; hepatocellular necrosis; jaundice; hepatitis	[33]
simvastatin	SN	statin	negative control	CYP3A4	ALT/AST elevations; jaundice; hepatitis	[34, 35]
troglitazone	TGZ	antidiabetic	positive control	CYP1A1; 2C8; 2C19; 3A4	fulminant hepatitis; ALF	[36, 37]

Table S2. Drugs used in this study and their reported adverse effects on the liver.

Reference List

1. Lu, J., et al., *Amiodarone exposure during modest inflammation induces idiosyncrasy-like liver injury in rats: role of tumor necrosis factor-alpha.* Toxicol Sci, 2012. **125**(1): p. 126-33.
2. Pollak, P.T. and S.L. Shafer, *Use of population modeling to define rational monitoring of amiodarone hepatic effects.* Clin Pharmacol Ther, 2004. **75**(4): p. 342-51.
3. Stamper, B.D., et al., *Differential regulation of mitogen-activated protein kinase pathways by acetaminophen and its nonhepatotoxic regioisomer 3'-hydroxyacetanilide in TAMH cells.* Toxicol Sci, 2010. **116**(1): p. 164-73.
4. Halmes, N.C., et al., *The acetaminophen regioisomer 3'-hydroxyacetanilide inhibits and covalently binds to cytochrome P450 2E1.* Toxicol Lett, 1998. **94**(1): p. 65-71.
5. Jaeschke, H., et al., *Acetaminophen hepatotoxicity and repair: the role of sterile inflammation and innate immunity.* Liver Int, 2012. **32**(1): p. 8-20.
6. Manyike, P.T., et al., *Contribution of CYP2E1 and CYP3A to acetaminophen reactive metabolite formation.* Clin Pharmacol Ther, 2000. **67**(3): p. 275-82.
7. Pirmohamed, M., S. Madden, and B.K. Park, *Idiosyncratic drug reactions. Metabolic bioactivation as a pathogenic mechanism.* Clin Pharmacokinet, 1996. **31**(3): p. 215-30.
8. Daly, A.K., *Using genome-wide association studies to identify genes important in serious adverse drug reactions.* Annu Rev Pharmacol Toxicol, 2012. **52**: p. 21-35.
9. Phillips, E.J. and S.A. Mallal, *HLA-B*1502 screening and toxic effects of carbamazepine.* N Engl J Med, 2011. **365**(7): p. 672; author reply 673.
10. Bjornsson, E., *Hepatotoxicity associated with antiepileptic drugs.* Acta Neurol Scand, 2008. **118**(5): p. 281-90.
11. Syn, W.K., et al., *Carbamazepine-induced acute liver failure as part of the DRESS syndrome.* Int J Clin Pract, 2005. **59**(8): p. 988-91.
12. McKnight, C., H. Guirgis, and N. Votolato, *Clozapine rechallenge after excluding the high-risk clozapine-induced agranulocytosis genotype of HLA-DQB1 6672G>C.* Am J Psychiatry, 2011. **168**(10): p. 1120.
13. Dragovic, S., et al., *Role of human glutathione s-transferases in the inactivation of reactive metabolites of clozapine.* Chem Res Toxicol, 2010. **23**(9): p. 1467-76.
14. Damsten, M.C., et al., *Application of drug metabolising mutants of cytochrome P450 BM3 (CYP102A1) as biocatalysts for the generation of reactive metabolites.* Chem Biol Interact, 2008. **171**(1): p. 96-107.
15. Valevski, A., et al., *HLA-B38 and clozapine-induced agranulocytosis in Israeli Jewish schizophrenic patients.* Eur J Immunogenet, 1998. **25**(1): p. 11-3.
16. Hummer, M., et al., *Hepatotoxicity of clozapine.* J Clin Psychopharmacol, 1997. **17**(4): p. 314-7.
17. Fredriksson, L., et al., *Diclofenac inhibits tumor necrosis factor-alpha-induced nuclear factor-kappaB activation causing synergistic hepatocyte apoptosis.* Hepatology, 2011. **53**(6): p. 2027-41.

18. Deng, X., et al., *Inflammatory stress and idiosyncratic hepatotoxicity: hints from animal models.* Pharmacol Rev, 2009. **61**(3): p. 262-82.
19. Boelsterli, U.A., *Diclofenac-induced liver injury: a paradigm of idiosyncratic drug toxicity.* Toxicol Appl Pharmacol, 2003. **192**(3): p. 307-22.
20. Daly, A.K. and C.P. Day, *Genetic association studies in drug-induced liver injury.* Drug Metab Rev, 2012. **44**(1): p. 116-26.
21. Srivastava, A., et al., *Role of reactive metabolites in drug-induced hepatotoxicity.* Handb Exp Pharmacol, 2010(196): p. 165-94.
22. Zand, R., et al., *Inhibition and induction of cytochrome P4502E1-catalyzed oxidation by isoniazid in humans.* Clin Pharmacol Ther, 1993. **54**(2): p. 142-9.
23. Lin, C.L., et al., *Unexpected emergence of acute hepatic injury in patients treated repeatedly with ketoconazole.* J Clin Gastroenterol, 2008. **42**(4): p. 432-3.
24. Kim, T.H., et al., *Liver cirrhosis developed after ketoconazole-induced acute hepatic injury.* J Gastroenterol Hepatol, 2003. **18**(12): p. 1426-9.
25. Bernuau, J., F. Durand, and D. Pessayre, *Ketoconazole-induced hepatotoxicity.* Hepatology, 1997. **26**(3): p. 802.
26. Aithal, G.P., *Hepatotoxicity related to antirheumatic drugs.* Nat Rev Rheumatol, 2011. **7**(3): p. 139-50.
27. West, S.G., *Methotrexate hepatotoxicity.* Rheum Dis Clin North Am, 1997. **23**(4): p. 883-915.
28. Xu, J.J., et al., *Cellular imaging predictions of clinical drug-induced liver injury.* Toxicol Sci, 2008. **105**(1): p. 97-105.
29. Stewart, D.E., *Hepatic adverse reactions associated with nefazodone.* Can J Psychiatry, 2002. **47**(4): p. 375-7.
30. Ali, S., J.D. Pimentel, and C. Ma, *Naproxen-induced liver injury.* Hepatobiliary Pancreat Dis Int, 2011. **10**(5): p. 552-6.
31. Czaja, A.J., *Drug-induced autoimmune-like hepatitis.* Dig Dis Sci, 2011. **56**(4): p. 958-76.
32. Boelsterli, U.A., et al., *Bioactivation and hepatotoxicity of nitroaromatic drugs.* Curr Drug Metab, 2006. **7**(7): p. 715-27.
33. Blum, A., *Ofloxacin-induced acute severe hepatitis.* South Med J, 1991. **84**(9): p. 1158.
34. Bjornsson, E., E.I. Jacobsen, and E. Kalaitzakis, *Hepatotoxicity associated with statins: reports of idiosyncratic liver injury post-marketing.* J Hepatol, 2012. **56**(2): p. 374-80.
35. Law, M. and A.R. Rudnicka, *Statin safety: a systematic review.* Am J Cardiol, 2006. **97**(8A): p. 52C-60C.
36. Jaeschke, H., *Troglitazone hepatotoxicity: are we getting closer to understanding idiosyncratic liver injury?* Toxicol Sci, 2007. **97**(1): p. 1-3.
37. Kaplowitz, N., *Idiosyncratic drug hepatotoxicity.* Nat Rev Drug Discov, 2005. **4**(6): p. 489-99.
